# Response to: “Addressing Immortal Time Bias in Estimating the Effect of Prophylactic Antiseizure Medication After Lobar Intracerebral Hemorrhage”

**DOI:** 10.1007/s12028-026-02642-9

**Published:** 2026-09-08

**Authors:** Margaret Banker, Andrew M. Naidech

**Affiliations:** 1 Department of Preventive Medicine, Northwestern University Feinberg School of Medicine, Chicago, IL 60611 USA; 2 Department of Neurology, Northwestern University Feinberg School of Medicine, Chicago, IL 60611 USA

We thank the authors for their interest in and commentary on our paper [1]. The authors suggest that classification into the prophylaxis group required patients to remain seizure-free from symptom onset until hospital presentation and treatment initiation, and early seizures occurring in that window could classify patients in the no-prophylaxis group.

We prospectively recorded the time of symptom onset and presentation to our facility or a referring facility. The median time from symptom onset to initial presentation to either the referring facility or our facility was 4.3 h (1.1–11.5 h). This is a relatively brief interval during which an early seizure may have occurred without potential for treatment initiation.

We appreciate that the authors suggested additional methods, however, these would require the time of the first seizure, which was not available. Of note, time of onset is an NIH Common Data Element for stroke, but time of seizure is not a common data element for epilepsy, so this was not in the case report forms. We agree that time of the first dose and seizure could be helpful to allow a target trial emulation analysis with clone-censor weighting, and this might be helpful for future research.

We performed sensitivity analyses to try and address the author’s concerns. The potential misclassification could arise either because (i) a seizure occurred almost immediately after symptom onset, before the patient could reach the hospital and be offered treatment, or (ii) because a seizure occurred after some delay, but the patient arrived too late for a treatment decision to be made in time.

To address the first concern, we repeated the analysis excluding patients who presented very quickly after symptom onset (within 1, 2, or 4 h). This exclusion removes patients who could have experienced an immediate seizure before possibility of prophylaxis. We found similar results (Table 1), suggesting that this potential source of bias is not driving the observed association. To address the second concern, that a longer interval between symptom onset and hospital presentation could allow more time for an early seizure to occur before a treatment decision could be made, we repeated the analysis, excluding patients who presented later (after 6, 8, or 10 h). We again found similar results (Table 1); the direction and magnitude of effect seem robust to excluding patients who do not present within a few hours of ICH symptom onset.

**Table 1:** Sensitivity analyses excluding patients by time from symptom onset to hospital presentation

Analysis	aOR	95% CI
Primary analysis	0.25	(0.11–0.55)
*Excluding early presenters*		
Excluded ≤ 1 h	0.28	(0.11–0.69)
Excluded ≤ 2 h	0.26	(0.09–0.76)
Excluded ≤ 4 h	0.29	(0.09–0.98)
*Excluding later presenters*		
Excluded ≥ 6 h	0.19	(0.07–0.54)
Excluded ≥ 8 h	0.21	(0.08–0.56)
Excluded ≥ 10 h	0.22	(0.08–0.57)

In addition, we performed a quantitative bias analysis to determine the proportion of inaccurately assigned patients that would change our findings. The adjusted association remained until approximately 40% of patients with seizures who were classified as not receiving prophylaxis were hypothetically reclassified as having received prophylaxis, at which point the adjusted odds ratio crossed the null. Thus, our results are robust.

We appreciate the call for a prospective, randomized trial of prophylactic antiseizure medication, however, this seems unlikely to be performed. We proposed such a trial (Seizure Prophylaxis in Lobar ICH Treatment, SPLIT). However, it was not given sufficient priority for funding. The PEACH trial was conducted, but halted due to low recruitment [2]. It seems that future research will focus on identifying the patients most likely to have a subsequent seizure. Our data suggest that acute, lobar ICH is one reasonable criterion to consider antiseizure prophylaxis.

## Artificial Intelligence

AI was not used in any part of this manuscript.
